# Adolescents' Long‐Term Identity Tension After Politicized Cyber Shaming: Peer Support and Behavioural Visibility Management As Joint Conditions

**DOI:** 10.1002/jad.70207

**Published:** 2026-06-17

**Authors:** Linsen Yang

**Affiliations:** ^1^ The University of Sydney, School of Languages and Cultures Sydney Australia

**Keywords:** adolescence, China, identity tension, peer support, politicized cyber shaming, visibility management

## Abstract

**Introduction:**

Politicized cyber shaming recodes adolescents' cultural interests as evidence of political loyalty or moral belonging. This study examines how such shaming is associated with longer‐term identity tension in adolescence, focusing on public expression, peer belonging, and self‐evaluation after politicized online controversy.

**Methods:**

Data were collected in China in 2023 using an event‐anchored retrospective mixed‐methods design. The study combines front‐end data from Baidu Tieba, a Chinese topic‐based online forum, during the 2012 Diaoyu/Senkaku Islands dispute and the 2016 Terminal High Altitude Area Defense (THAAD) controversy, with a retrospective survey of 84 participants exposed as adolescents and timeline interviews with 18 participants. At event exposure, participants were 14–18 years old (M = 16.06, SD = 1.40), and 63.1% were women.

**Results:**

The controversy windows showed higher semantic hostility and first‐screen nationalist dominance than historical baselines. Adaptation was organised into limited expression, circle contraction, and public silence. Public silence showed the highest long‐term identity tension and perceived negative impact, circle contraction was intermediate, and limited expression was lowest. Peer support was associated with lower identity tension overall, behavioural visibility management with higher identity tension, and their interaction indicated joint association with adaptation.

**Conclusions:**

Politicized cyber shaming should be understood as an identity‐relevant social‐evaluative experience in adolescence. Support may reduce isolation, but it does not simply cancel harm when continued participation requires sustained visibility management.

1

When adolescents share popular culture or join fan communities on digital platforms, they also use visible interaction to explore identity and build peer belonging (Avci et al. 2025; Pérez‐Torres 2024). In politicized online environments, everyday interests can be reread as evidence of weak national loyalty or flawed moral standing. During the 2016 Terminal High Altitude Area Defense (THAAD) controversy over a U.S. missile‐defense system in South Korea, which China strongly opposed on the grounds that the system's radar could threaten its strategic security, the phrase “no idols before the nation” drew Hallyu (the Korean Wave) fan activity into public tests of loyalty, exposing ordinary participants to accusations of being unpatriotic. This article, therefore, asks how politicized public shaming of adolescents' everyday interests enters their developing boundaries of expression, peer belonging, and self‐understanding, and how it becomes a longer‐term identity tension.

Such pressure is embedded in Chinese online space, where state discourse, platform governance, and user practice shape a local nationalist digital ecology (Zhang et al. 2024; Chen et al. 2025b). Chinese‐style cancel culture, fan reporting, and digital nationalist mobilisation show that cultural interests can be placed in a frame of patriotism and disloyalty (Wang 2022; Tan and Li 2025). I refer to this process as politicized cyber shaming: visible online behaviour is interpreted as a breach of national‐political norms or group moral boundaries and then met with public blame, reporting, or expressive discipline. Shaming is deliberate here. Unlike cyberbullying or harassment, it foregrounds public moral evaluation, placing the target under visible judgement and making hiding, distancing, or visibility management plausible responses (Billingham and Parr 2020; Schmader and Lickel 2006).

Existing work provides a foundation but not a full account. Cyberbullying research links online victimisation to depression, lower self‐esteem, and internalising problems (Kowalski et al. 2014; Li et al. 2026). Public shaming research shows how collective harassment can narrow expression and produce self‐censorship (Forestal 2024; Penney 2020). Yet these literatures do not fully explain how politicized shaming becomes embedded in adolescents' longer‐term adaptation. Digital communication research treats platforms as sites where identity is performed and negotiated (Cover 2023). Every day shame also describes wanting to belong while feeling one “cannot fit in” (Probyn 2004, 345). These accounts matter for adolescence because they connect public evaluation to the ordinary work of being recognisable to others while still maintaining a coherent sense of self. The issue is therefore not only whether politicized shaming produces distress, but how it affects adolescents' self‐continuity and sense of safe presence in a community or public space (Sedikides et al. 2023).

To address this question, the study focuses on two windows in which cross‐border cultural participation became entangled with nationalist controversy on Baidu Tieba: the 2012 Diaoyu/Senkaku Islands dispute and the 2016 THAAD controversy. These windows are useful not because they represent all Chinese digital life, but because they make unusually visible the moment when cultural participation, peer judgement, and nationalist evaluation converge. For adolescents learning to maintain peer relations and understand the relation between self and collective, such experiences may become identity‐relevant social evaluation. The study uses an event‐anchored retrospective mixed‐methods design to identify adaptation pathways, compare identity tension and negative impact, and estimate how peer support and behavioural visibility management are jointly associated with identity tension.

## 2 Literature Review

### 2.1 From Digital Harm to Politicized Cyber Shaming

Cyberbullying research helps explain adolescent online victimisation, but it does not capture all forms of online harm. Digital hate research shows that cyberbullying, hate speech, harassment, and incivility overlap, while cyberhate and bias‐based cyberaggression emphasise attacks organised around social categories: targets may be groups or individuals classified by group identity or political orientation (Bedrosova et al. 2025; Jiménez‐Díaz and Del Rey 2025; Matthes et al. 2025). This distinction is important because the harm does not begin only from a dyadic conflict between an aggressor and a victim. It also emerges when a visible act is used to place someone inside a suspect category. The issue is therefore not only that someone is attacked online; it is that attackers use social categories to decide who can be degraded, excluded, or shamed.

Online public shaming literature explains how group categorisation becomes public punishment and norm enforcement. Shaming usually occurs when individuals are perceived to have violated social norms, moral standards, or public expectations. Its core is public moral criticism that marks a norm violator, punishes the target, warns bystanders, and reinforces group boundaries (Billingham and Parr 2020; Chen et al. 2025a; Forestal 2024). On social media, this process can link to digital vigilantism, collective harassment, and mob censorship, compressing expressive space through naming, surveillance, reporting, and amplified visibility (Penney 2020; Trottier 2017; Waisbord 2020).

Shaming, therefore, needs separate conceptual work. Aggression, bullying, and harassment describe hostile contact, repeated harm, or unequal power; shaming foregrounds a public moral‐affective process. The target is placed under evaluative gaze and marked as transgressive or out of place. Probyn's account of everyday shame links selfhood to place and ordinary belonging, especially the tension of wanting to belong while being unable to fit in (Probyn 2004). Psychological research similarly distinguishes shame from guilt: shame is more closely linked to avoidance, distancing, and image threat, whereas guilt is more linked to repair or approach (Lickel et al. 2005; Lickel et al. 2011; Schmader and Lickel 2006). Online shaming is thus not merely an attack. It creates a social position that may require hiding, distancing, or renewed self‐presentation management.

Research on Chinese digital nationalism and fan culture provides the context for this problem. Young Chinese netizens, fan culture, and popular idols can become involved in nationalist mobilisation, while boys' love (BL; danmei, Chinese‐language male‐male romance and fan fiction) fandom has long negotiated censorship, reporting, heteronormative norms, and moral suspicion (Fang and Repnikova 2018; Tian 2020; Xia 2024). Event‐based studies show that fan conflicts can be amplified through reporting mechanisms and moralised accusations. Foreign cultural products may be framed as cultural invasion, and supporters may pre‐emptively declare political stances to avoid attack or censorship (Tan and Li 2025; Wang 2022; Wang and Ge 2023; Zhang et al. 2025). These studies show why cultural preference can become publicly risky, but they less often follow what happens after the controversy has passed. The remaining question is how public shaming continues to shape expression, belonging, and identity judgement after adolescents' cultural interests are reread as a political stance or national loyalty.

### 2.2 Adolescent Digital Identity and Long‐Term Identity Tension

This form of online harm needs to be understood within adolescent development because it occurs when identity exploration, peer recognition, and social‐evaluative cues become salient. Reviews of social media and adolescent identity discuss identity exploration, commitment, self‐concept clarity, and identity distress, and suggest that these dimensions are tied more closely to platform participation than to time online alone (Avci et al. 2025). Digital environments participate in identity processes through choice, self‐presentation, audience feedback, and social comparison (Soh et al. 2024). Cover (2023) similarly conceptualises digital communication as a site where identity is performed, recognised, and negotiated. When public shaming changes how an expression is recognised, it can affect how adolescents present and understand the identities attached to that expression.

Social media can also operate as a digital social mirror. Adolescents express interests, views, and beliefs on platforms and use self‐presentation, social comparison, role models, and audience feedback to understand themselves (Pérez‐Torres 2024). Authentic self‐presentation is often associated with higher self‐concept clarity, whereas comparison experiences may relate to identity exploration and distress (Avci et al. 2025; Pérez‐Torres 2024). Self‐continuity research adds a temporal dimension: lower or threatened self‐concept clarity may weaken the subjective connection among past, present, and future selves (Jiang et al. 2020; Sedikides et al. 2023).

The developmental features of adolescence make such digital evaluation consequential without implying uniform vulnerability. Adolescence may be a sensitive period for sociocultural processing, and peer influence operates through how adolescents encode, interpret, and respond to social cues (Cheng et al. 2024; Venticinque et al. 2024). When valued aspects of the self are judged negatively, or expected to be judged negatively, the situation can constitute social‐evaluative threat (Silk et al. 2024). Persistent content and quantifiable feedback may intensify responses to peer judgement (Nesi et al. 2018a, 2018b; Orben et al. 2024). These strands of work point to a common developmental problem: adolescents are not only reacting to hostile content, but also learning what kinds of expression remain recognisable, valued, or safe. What remains insufficiently clear is how politicized moral evaluation of adolescents' interests is related to identity exploration, self‐concept clarity, peer belonging, and self‐continuity over time. The study, therefore, treats long‐term identity tension as friction around public expression, peer belonging, and self‐evaluation after politicized cyber shaming, rather than as traditional identity status or general self‐esteem change.

### 2.3 Platform Visibility, Peer Support, and Behavioural Visibility Management

Platform visibility structures help explain why online evaluation may remain part of adolescents' social environments after an event. Social media affordances such as persistence, publicness, 24‐h availability, and quantifiable feedback change adolescent peer interaction, making evaluation, victimisation, and status cues easier to preserve, re‐view, spread, and translate into visible feedback (Nesi et al. 2018a, 2018b; Orben et al. 2024). Recent work also rejects treating social media use as a unitary exposure and emphasises content, functions, platform features, and individual differences. Visible approval signals may strengthen the social reward value of hostile expression (Maheux et al. 2025; Vaid et al. 2024; Walther 2022, 2022; [Bibr jad70207-bib-0046]).

Platform conditions, however, do not automatically produce the same consequences, and adolescents are not passive recipients of digital environments. Positive social connections and peer relationships are important resources for youth mental health. Online peer support can provide understanding and belonging through emotional support, shared experience, and community environments (Birrell et al. 2025; Yuan et al. 2025). Yet it may also involve triggering content, emotional exhaustion, and relational pressure. This duality is important for the present study because support can make withdrawal less necessary while also keeping adolescents connected to the very spaces in which boundary negotiation continues. Peer support is therefore better understood as a relational condition than as a one‐directional protective variable (Yuan et al. 2025).

A related body of work concerns audience boundaries and visibility regulation. Research on networked privacy and context collapse shows that privacy on social media is a continuously negotiated information boundary; adolescents manage who can see information and how different audiences are distinguished while participating in peer networks (Marwick and boyd 2014; Vitak 2012). Users also regulate visibility through audience grouping, privacy settings, self‐censorship, private messaging, multiple accounts, and platformed self‐presentation (Duffy and Chan 2019; Vitak et al. 2015). In ordinary circumstances, these practices can be read as privacy or impression‐management strategies. After politicized shaming, however, they also become ways of handling the possibility that an interest, name, or trace will be reclassified. What remains insufficiently clear is how relational resources and audience‐boundary regulation are jointly associated with adaptation differences in highly visible and spreadable online evaluative contexts. This gap provides the basis for examining peer support and behavioural visibility management together.

### 2.4 Research Questions and Theoretical Expectations

The article does not assume that politicized cyber shaming produces a single, homogeneous outcome. It examines how adolescents form different longer‐term modes of adaptation in expression, belonging, and self‐evaluation. Adaptation here does not mean generic platform‐use change; it means longer‐term social‐evaluative adaptation within adolescent identity development. This framing keeps the empirical questions close to the developmental problem: whether adolescents continue to speak, narrow the spaces in which they speak, or become publicly silent after a shaming event, and how those choices are related to identity tension. The study asks: RQ1, what identifiable pathways of adaptation emerge after politicized cyber shaming? RQ2, do these pathways differ in long‐term identity tension and overall negative impact? RQ3, how are peer support and behavioural visibility management, separately and jointly, associated with long‐term identity tension?

Based on the literature reviewed above, the study advances three theoretical expectations: H1, peer support is negatively associated with long‐term identity tension; H2, behavioural visibility management is positively associated with long‐term identity tension; and H3, peer support moderates the association between behavioural visibility management and long‐term identity tension. Specifically, this positive association is expected to be stronger when peer support is higher and weaker when peer support is lower. The logic is not that support is harmful or that management itself causes harm, but that support may sustain continued participation while management often reflects ongoing boundary work.

## 3 Methods

### 3.1 Research Design, Platform, and Sample

This study uses an event‐anchored retrospective mixed‐methods design, integrating front‐end environmental data, an event‐anchored retrospective survey, and timeline interviews. The methodological choices were adapted from event‐based digital nationalism research, platform‐visibility work, scale‐development guidance, and mixed‐methods research to fit the present questions and data structure. Retrospective survey and interview data were collected in China in 2023. The design does not estimate causal effects; it describes the visible platform environment in which politicized cyber shaming occurred and analyzes how participants retrospectively organised their adaptation. Event anchoring limited platform materials, survey responses, and interviews to two controversy windows rather than to general memories of social media use. This was necessary because the study asks about adaptation after particular public controversies, not about average platform exposure. Front‐end data establishes the visible climate around the focal events, survey data compare patterned differences across participants, and timeline interviews explain how participants connected those events to later participation, withdrawal, support, and boundary work. Because the survey and interviews included reports made years later, individual‐level findings are interpreted as conditional associations and retrospective meaning construction.

The study focuses on relevant interest communities in Baidu Tieba, a Chinese topic‐based online forum. Tieba is organised around topics or interests and includes public posts, searchable archives, reply ordering, and visible interaction, making it suitable for observing visible discourse environments during controversies. These platform features also matter substantively: public threads and searchable archives make accusations easier to revisit, while reply ordering and opening‐screen content shape what participants see first when entering a discussion. The two focal windows are the 2012 Diaoyu/Senkaku Islands dispute and the 2016 THAAD controversy. The latter dispute triggered nationalist criticism of South Korean cultural consumption online. The two windows are treated as highly salient nationalist controversies rather than representative samples of Chinese online nationalism, and were selected to observe how nationalist evaluation, public shaming, and post‐event adaptation concentrate under similar Table 1 and Table 2 platform logics (Zhang et al. 2024; Huang 2023; Wang 2022).

**Table 1:** Controversy‐window visibility pressure relative to historical baselines.

Indicator	Baseline	Event	Welch test
Semantic hostility (Esem)	0.37%	2.41%	*t* = 12.40, *p* < 0.001
First‐screen nationalist dominance (Evis)	65.50%	87.86%	*t* = 8.90, *p* < 0.001

*Note:* Indicators are descriptive proxies of the front‐end visible environment and do not represent platform back‐end ranking mechanisms or causal exposure. Page retrieval, baseline construction, retrieval success, and the auxiliary environmental model are reported in Supporting Appendix C.

**Table 2:** Adaptation pathways, core outcomes, and behavioural validation indicators.

Path	PS M (SD)	VM‐B M (SD)	ICI* M (SD)	IMP M (SD)	Tieba use
Public silence	1.72 (0.45)	3.38 (0.37)	6.67 (0.17)	5.00 (0.00)	77.8% fully left
Circle contraction	2.41 (0.40)	6.24 (0.68)	5.83 (0.69)	4.45 (0.51)	84.8% substantially reduced
Limited expression	5.99 (0.73)	5.81 (0.47)	3.99 (0.96)	2.92 (0.93)	75.0% no substantial change

*Note:* Complete pairwise contrasts, adjusted *p*‐values, effect sizes, and distributional checks are reported in Supplementary Appendix C.

The survey sample included 84 respondents who had been active in relevant Tieba interest communities during the focal windows and reported politicized shaming or closely related hostility. The sample captures theoretically relevant exposed cases rather than population prevalence. Women accounted for 63.1%; the mean age at event exposure was 16.06 years (SD = 1.40, range = 14–18). The age‐at‐exposure framing is central because the study concerns adolescent experience, even though respondents completed the survey as young adults after the historical events; the recall interval was approximately 11 years for the 2012 cohort and 7 years for the 2016 cohort. Timeline interviews included 18 participants selected to maximise variation in adaptation style, support conditions, and visibility‐management practices. Recruitment, inclusion criteria, and sample information are reported in Supplementary Appendix B.

### 3.2 Indicators, Measures, and Analytic Strategy

Front‐end environmental data came from publicly visible Tieba pages in the two controversy windows rather than solely from participants' retrospective reports. The study constructed two page‐level descriptive indicators. Semantic hostility (Esem) refers to the density of politicized derogatory labels and hostile expressions in visible page text. First‐screen nationalist dominance (Evis) refers to the proportion of items visible without scrolling that could be coded as expressing a nationalist stance. Esem used a study‐specific contextual stigma dictionary, and Evis was manually stance‐coded. These indicators are not intended to reconstruct platform back‐end ranking or individual causal exposure. They instead describe the front‐end environment that a participant could encounter when entering archived or active discussions. Because online harassment and digital hate detection are highly context dependent, a contextualised dictionary is more appropriate here than a generic toxicity dictionary (Rezvan et al. 2020; Matthes et al. 2025). Dictionary construction, coding rules, reliability, and validation results are reported in Supplementary Appendices A and C.

The core survey indicators were behavioural visibility management (VM‐B), peer support (PS), and the Identity Conflict Index specification for long‐term identity tension (ICI*). All three are treated as study‐specific, event‐sensitive composite indicators rather than fully validated general psychological scales. Item construction followed the principles of construct definition, content‐domain specification, and transparent item provenance (Boateng et al. 2018; Clark and Watson 2019). VM‐B was measured by three items capturing practices such as alternative accounts, differentiated screen names, and audience restriction. PS was measured by three items covering post‐event emotional support seeking, standing with other targeted fans, and perceived solidarity. ICI* is not a traditional identity‐status measure (Marcia 1966), but an event‐after composite indicator capturing long‐term tension in public expression, peer belonging, and self‐evaluation. This distinction is important because the study does not classify respondents into identity statuses; it asks whether politicized public evaluation is associated with later friction in how adolescents present themselves, locate belonging, and evaluate the self. Items, scoring rules, reliability, and the rationale for excluding ICI12 are reported in Supporting Appendix A.

Pathway labels are analytical groupings based on prespecified thresholds, used to organise heterogeneity in post‐event adaptation; they are not latent classes or naturally existing psychological types. Based on VM‐B and PS, participants were classified into limited expression, circle contraction, and public silence. The labels were chosen to describe observable combinations of continued participation, narrowing of interaction, and withdrawal from public expression. Thresholds and robustness checks are reported in Supplementary Appendix C. Quantitative analysis compared front‐end environmental indicators with historical baselines, compared pathways in core outcomes and behavioural validation indicators, and used ordinary least squares models to estimate the conditional associations of PS, VM‐B, and their interaction with ICI*. Because the sample was modest, models used HC3 robust standard errors. Results are interpreted as exploratory and associational. Interview materials were coded around visibility pressure, audience‐boundary work, peer‐support dynamics, and identity tension to explain pathway differences and interaction patterns. Integration occurred at the interpretation stage: quantitative patterns identified where adaptation differed, while interviews clarified how respondents understood continued presence, retreat, or silence over time.

## 4 Results

Compared with historical baselines, both semantic hostility and first‐screen nationalist dominance increased significantly during the two controversy windows. Esem increased from 0.37% to 2.41%, approximately 6.5 times the baseline; Evis increased from 65.50% to 87.86%. Both Welch tests reached *p* < 0.001. These results indicate that the focal events did not occur in neutral discussion environments, but in front‐end platform environments with elevated visible hostility and nationalist opinion climate. The environmental indicators, therefore, provide the descriptive context for interpreting later self‐reports: participants were recalling adaptation after periods in which politicized hostility and visible nationalist dominance were materially higher than baseline conditions.

Pathway results showed that limited expression, circle contraction, and public silence were not the same response at different degrees of severity, but three modes of adaptation formed under different combinations of relational resources, boundary management, and platform behaviour. Public silence combined the lowest peer support with lower active management, circle contraction combined lower support with the highest management, and limited expression combined higher support with higher management. ICI* showed a clear gradient: public silence was highest (M = 6.67), circle contraction was intermediate (M = 5.83), and limited expression was lowest (M = 3.99). Overall negative impact followed the same direction, but public silence showed ceiling concentration on IMP; related inferential tests are therefore in the supplementary materials. Behavioural indicators supported the pathway labels: 77.8% of public silence fully left Tieba after the event, 84.8% of circle contraction substantially reduced use, and 75.0% of limited expression reported no substantial change. The intermediate position of circle contraction is especially informative. It suggests that retreat into narrower circles can preserve some relational connection while requiring intensive boundary work. These behavioural patterns are important because they show that identity tension was not simply an attitude detached from later practice. It was linked to whether participants stayed visible, narrowed interaction, or left public posting altogether.

The regression model further showed that peer support was negatively associated with long‐term identity tension (B = −0.665, *p* < 0.001), behavioural visibility management was positively associated with long‐term identity tension (B = 0.339, *p* = 0.012), and their interaction was also significant (B = 0.281, *p* = 0.002). This interaction indicates that support and management are not two independent conditions. When support was lower, the association between visibility management and identity tension was weaker and closer to a low‐management state after exit or silence. When support was higher, the positive association between management and identity tension was stronger. This pattern is consistent with the interview accounts: support could make continued participation emotionally possible, but continued participation also required respondents to keep managing audiences, names, accounts, and the risk of renewed judgement. The finding, therefore, helps interpret why support reduced identity tension overall while not eliminating the costs of remaining visible. Complete pairwise contrasts, full covariate models, distributional checks, and robustness tests are reported in Supplementary Appendix C.

## 5 Discussion

### 5.1 From Digital Harm Consequences to Identity Adaptation Pathways

These findings show that adolescent adaptation after politicized cyber shaming cannot be reduced to psychological harm or recovery‐versus‐withdrawal. Under visible pressure, adaptation differentiated into limited expression, circle contraction, and public silence. Shaming matters because it marks the target as a norm violator and positions them as out of place (Probyn 2004). Shame research links threats to self‐image or group image with avoidance, distancing, and image protection (Lickel et al. 2005; Lickel et al. 2011; Schmader and Lickel 2006). Public silence makes this logic visible. It can reduce renewed attacks, screenshots, reporting, and being watched, but it does not prove that harm has ended. Withdrawal and self‐censorship are often defensive responses to unpredictable attack, not evidence that expression has become safe (Waisbord 2020; Penney 2020; Zhang et al. 2025). In this study, public silence is a continuing identity defence: it lowers visibility, but also weakens public routes to peer feedback and identity validation. This helps explain why the highest identity tension appeared in the pathway with the least public expression. Silence protected participants from renewed exposure, but it also left the conflict between interest, belonging, and public recognisability unresolved.

Long‐term identity tension is not traditional identity status or general self‐esteem change. It refers to persistent friction around expression, belonging, and self‐evaluation after shaming. Digital identity research shows that interest expression and audience feedback help adolescents understand the self, while self‐continuity research emphasises intelligible links across past, present, and future self‐experience (Avci et al. 2025; Pérez‐Torres 2024; Soh et al. 2024; Jiang et al. 2020; Sedikides et al. 2023). Cover (2023) conceptualises digital identity as performed, recognised, and negotiated through online interaction. Politicized cyber shaming changes the conditions of that process: platform experiences that once helped adolescents express interests and confirm the self are moved into a frame of political stance and moral belonging. Long‐term identity tension, therefore, reflects self‐continuity friction when interests and expressions are publicly redefined. The contribution is not to claim that a single event determines identity development, but to show how a public evaluative episode can become part of the conditions under which adolescents later decide what can be shown, where belonging can be trusted, and how a valued interest can be integrated into the self.

### 5.2 How Relational Resources and Visibility Boundaries Organise Adaptation

The interaction results show that post‐shaming adaptation is not determined by a single protective or risk factor. Peer support was associated with lower identity tension, indicating relational resources of being understood, received, and still belonging (Birrell et al. 2025; Yuan et al. 2025). But support does not simply cancel harm. Under higher support, the positive association between behavioural visibility management and identity tension was stronger; under lower support, this association weakened and resembled a low‐management state after exit or silence. Peer affirmation can provide identity validation while also keeping adolescents connected to environments where social evaluation remains possible. This is why support has to be interpreted developmentally rather than only clinically. It can reduce isolation and make continued participation imaginable, but continued participation also means that adolescents must keep negotiating how visible they can be. In politicized settings, sustaining belonging may require continuing boundary regulation.

Behavioural visibility management should also be understood as continuing boundary work rather than simply as coping with success or failure. Adolescents use alternative accounts, differentiated screen names, audience restriction, or movement across spaces to balance participation and risk. Networked privacy and context collapse research shows that audience boundaries on social media require continuous negotiation (Marwick and boyd 2014; Vitak et al. 2015; Duffy and Chan 2019). After politicized cyber shaming, boundary management is not only an ordinary privacy choice. It becomes an identity practice for managing social‐evaluative threat: adolescents control digital traces and limit audiences to buffer the tension between belonging and renewed public labelling. This explains why higher management can coincide with higher identity tension. Management marks continued exposure to a problem that has not fully disappeared; it is the work required to keep an interest or peer tie alive under conditions of possible reclassification. This does not treat VM‐B as a direct measure of immediate shame; it shows that visibility management is both risk regulation and identity‐boundary regulation.

The study's contribution to adolescent development research is to define politicized cyber shaming as an identity‐relevant social‐evaluative experience rather than ordinary platform conflict or adult political communication. Adolescents encode and respond to evaluative information from peer and public environments, and this information enters judgements about behaviour, relationships, and the self (Cheng et al. 2024; Venticinque et al. 2024; Silk et al. 2024). When evaluation becomes politicized pressure, turning valued interests into public judgements of loyalty or moral belonging, digital harm can enter the long‐term process of adolescent identity adaptation. This developmental framing also clarifies why the study is not only about China or fandom. The Chinese cases make the mechanism visible, but the broader point is that politicized public evaluation can transform ordinary adolescent cultural participation into a problem of recognition, safety, and self‐understanding. The study, therefore, moves beyond a risk‐only account of online harm by showing how adolescents actively reorganise visibility, relationships, and self‐presentation after public judgement.

Chinese digital nationalism and fan politics provide a clear setting in which to observe this process. The contribution is not only to show that politicized shaming exists on Chinese platforms, but to show how public reclassification of adolescents' everyday interests can extend digital harm into longer‐term questions of expression, belonging, and self‐understanding. This matters for developmental science because the case is not merely an example of a local controversy. It shows how a national digital ecology can enter the ordinary peer and identity work of adolescence through cultural participation. The study, therefore, connects digital harm, digital identity, and adolescent social evaluation within a non‐WEIRD developmental context (Sirin et al. 2024).

### 5.3 Limitations

Several limitations should be noted. First, the event‐anchored retrospective design cannot eliminate recall bias, post‐hoc reconstruction, or the influence of current identity position on narratives of past experience. Second, the modestly theoretically sampled cases do not estimate prevalence or average effects for all Chinese adolescents. Third, the study did not directly measure immediate shame, guilt, humiliation, or anger during the attack. The shame literature is therefore used to interpret the social‐evaluative logic of shaming and possible avoidance or distancing motives, not as evidence that these mechanisms were directly tested. Fourth, Esem and Evis capture the front‐end visible environment rather than private messages, off‐platform spillovers, or back‐end recommendation mechanisms. The indicators should therefore be read as descriptive proxies of visible climate, not as full measures of exposure. Public silence also showed ceiling concentration on overall negative impact, and low VM‐B among leavers may partly reflect reduced applicability of active‐management items. These results should therefore be read as exploratory evidence of longer‐term adaptation rather than causal estimates.

Future research could use longitudinal designs, linked digital traces, cross‐platform comparison, and finer measurement of exit to examine how adaptation pathways unfold over time. Further work on how family support, school climate, offline peer relations, and platform governance interact with peer support and visibility management would also help integrate politicized digital harm into adolescent development research.

## 6 Conclusion

Politicized cyber shaming may leave adolescents with more than a memory of one online conflict. During identity exploration, visible hostility and possible re‐exposure can turn ordinary interests into continuing tests of recognition and loyalty. Longer‐term identity tension does not take a single form: peer support and behavioural visibility management jointly organise limited expression, circle contraction, and public silence. The central implication is that support, withdrawal, and boundary management are not separate endings to harm, but different ways adolescents try to preserve belonging and self‐understanding after public moral judgment. This is why the adolescent frame matters: the same online event is also a context in which young people learn what kinds of cultural attachment can be safely made public. Politicized digital harm should therefore be understood as a social‐evaluative experience embedded in adolescent identity development.

## Author Contributions

**Linsen Yang:** conceptualization, investigation, funding acquisition, writing – original draft, methodology, validation, visualization, writing – review and editing, project administration, formal analysis, software, data curation, supervision, resources.

## Funding

The author has nothing to report.

## Ethics Statement

This study involves human participants and was approved by The University of Sydney Human Research Ethics Committee (HREC; Approval No. 2023/HE000837). The study was conducted in accordance with the approved protocol.

## Conflicts of Interest

The author declares no conflicts of interest.

## Consent to Participate

All participants provided informed consent.

## Consent for Publication

Not applicable. The manuscript does not contain identifiable individual‐level information.

## Declaration of Generative AI Use

During preparation of this manuscript, the author used OpenAI ChatGPT (GPT‐5) and Qwen3 14B to assist with language editing. These tools were used to improve clarity, consistency, and submission formatting. The author reviewed, revised, and verified all AI‐assisted outputs and takes full responsibility for the final content.

## Supporting information

Supporting File

## Data Availability

De‐identified analysis data, codebooks, and minimal replication scripts are available on the Open Science Framework (OSF) at DOI: 10.17605/OSF. IO/6S4YK. Access is subject to the ethical restrictions governing participant privacy and the sensitive political nature of the materials.
