# Supplementary material for: Adolescents' Long‐Term Identity Tension After Politicized Cyber Shaming: Peer Support and Behavioural Visibility Management As Joint Conditions
Source: J Adolesc. 2026 Jun 17;98(6):2106–14. doi: 10.1002/jad.70207 (PMC13440694; doi:10.1002/jad.70207)
Supplement: Supplementary file 1 [file JAD-98-2106-s001.docx]

Supplementary Materials for

Adolescents’ Long-Term Identity Tension After Politicized Cyber Shaming:

Peer Support and Behavioural Visibility Management as Joint Conditions

# Appendix A. Measures, construct provenance, and scoring rules

## A1. Construct provenance and analytic role

**Table A1**

Construct Provenance and Analytic Role

| **Construct** | **Analytic role** | **Item IDs** | **Response anchors** | **Construction rule** | **Reliability / note** |
| --- | --- | --- | --- | --- | --- |
| Behavioural visibility management (VM-B) | Primary predictor | DIS01-DIS03 | 0 = missing; 1-7 Likert | Mean of 3 items when at least 2 are answered | α = .97; operationalises post-event boundary regulation via audience control and exposure reduction; among full leavers, low scores indicate limited active management after exit |
| Peer support (PS) | Primary predictor | CS01-CS03 | 0 = missing; 1-7 Likert | Mean of 3 items when at least 2 are answered | α = .99; combines emotional support seeking and solidarity |
| Long-term identity tension (ICI*) | Primary outcome | ICI01-ICI13, excluding ICI12 | 0 = missing; 1-7 Likert | Mean of 12 items when at least 7 are answered | α = .99; domains derived from digital identity, self-continuity, and social-evaluative accounts; interview-refined; majority-items threshold chosen a priori; ICI12 omitted from primary specification |
| ICI_Total | Robustness outcome | ICI01-ICI13 | 0 = missing; 1-7 Likert | Mean of 13 items when at least 7 are answered | α = .99; full-item robustness specification |
| Online-offline self-discrepancy | Validation indicator | DIS04 | 0 = missing; 1-7 Likert | Single item | Kept separate from primary ICI specification |
| Visibility-management cost | Validation indicator | DIS05-DIS06 | 0 = missing; 1-7 Likert | Mean of 2 items when at least 1 is answered | α = .97 |
| Overall negative impact (IMP) | Secondary outcome | IMP01-IMP02 | 0 = unable; 1-5 impact scale | Mean of 2 items when at least 1 is answered | Two global impact items; Pearson r = .81 (Spearman-Brown = .89) |
| Tieba use change (I1) | External validity indicator | I1 | 0 = unable; 1-3 categorical | Single item | 1 = completely left; 2 = substantially reduced; 3 = no significant change |

*Note. Table A1 summarises construct provenance. For the focal outcome, item domains drew on digital identity, self-continuity, and social-evaluative frameworks and were refined using recurring phrasing from timeline interviews. The primary ICI* scoring rule required at least seven of the 12 retained items so that the index remained based on a majority of items while retaining respondents whose retrospective pathways made some items less applicable. Additional survey modules not analysed in the present article are not included in the core scoring tables below.*

## A2. Core item wording used in the manuscript

Unless otherwise noted, 0 indicates “unable to recall or prefer not to answer” and is treated as missing in scored variables.

### A2.1 Long-term identity tension items (ICI01-ICI13)

**Table A2**

Long-Term Identity Tension Items (ICI01-ICI13)

| **Item** | **Wording** |
| --- | --- |
| ICI01 | I struggled with whether liking Japanese and Korean culture conflicts with being Chinese. |
| ICI02 | I worried that my enthusiasm for foreign culture would be seen as unpatriotic. |
| ICI03 | I did not dare talk openly about my fandom interests. |
| ICI04 | When criticised, I felt compelled to defend my preference. |
| ICI05 | I doubted whether continuing to support foreign idols was appropriate. |
| ICI06 | I avoided certain topics to reduce controversy. |
| ICI07 | I feared that others would question my cultural identity. |
| ICI08 | I considered distancing myself from the culture I liked. |
| ICI09 | Online criticism made me question my loyalty. |
| ICI10 | I felt restricted when expressing admiration for Japanese and Korean culture. |
| ICI11 | After criticism, I felt less connected to domestic peer groups. |
| ICI12 | My online and offline identities seemed different. |
| ICI13 | These experiences made me rethink who I am. |

*Note.* ICI12 was retained in the questionnaire but excluded from the primary ICI* specification because it overlaps conceptually with the separate online-offline self-discrepancy indicator (DIS04).

### A2.2 Support, management, and impact items

**Table A3**

Support, Management, and Impact Items

| **Item** | **Wording** |
| --- | --- |
| CS01 | I sought emotional support from like-minded friends. |
| CS02 | I stepped in to help other targeted fans. |
| CS03 | I felt a sense of solidarity with other fans. |
| DIS01 | I use different screen names in different forums to separate identities. |
| DIS02 | I created a dedicated account solely for fandom activities. |
| DIS03 | I restrict who can view my fandom content. |
| DIS04 | My online and offline selves appear different. |
| DIS05 | Switching identities makes me feel tired. |
| DIS06 | I often struggle to remember which identity I am using. |
| IMP01 | Overall, how much have these online attacks affected your mental health? |
| IMP02 | Overall, how much have these attacks affected your cultural identity? |
| I1 | After the focal period, my Tieba usage became: 1 = completely left; 2 = substantially reduced; 3 = no significant change; 0 = unable. |

*Note.* CS and DIS items use 1–7 Likert agreement scales. CS02–CS03 capture solidarity with other targeted fans. In the manuscript, DIS01–DIS03 define behavioural visibility management (VM-B), whereas DIS04 remains a separate online–offline self-discrepancy indicator. Among respondents who later withdrew almost entirely from public posting, low VM-B should be read as limited active management rather than as evidence that management items remained equally applicable after exit. IMP uses the scored 0–5 impact scale reported in the analysis-ready dataset.

## A3. Contextual stigma lexicon and first-screen coding validation

The contextual stigma lexicon is a semi-open dictionary developed for this study to identify politicized insults and derogatory labels salient in Tieba texts. It was assembled from platform keyword suggestions, labels observed in the 2012 and 2016 event-window corpora, and context-relevant prior work. After de-duplication, the lexicon contained 268 entries grouped into five categories. Because many entries are harmful slurs, the full lexicon is not reproduced in the public supplement; controlled access is retained for verification purposes.

**Table A4**

Contextual Stigma Lexicon and First-Screen Coding Validation

| **Metric** | **Value** |
| --- | --- |
| Cohen's kappa (nationalist / non-nationalist) | 0.812 |
| Cohen's kappa (three-class stance coding) | 0.840 |
| Krippendorff's alpha (nationalist / non-nationalist) | 0.810 |
| Krippendorff's alpha (three-class stance coding) | 0.840 |
| Lexicon precision | 0.931 |
| Lexicon recall | 0.850 |
| Lexicon F1 | 0.889 |
| Lexicon accuracy | 0.936 |

*Note.* Reliability refers to the manual first-screen stance coding sample. Validation metrics refer to dictionary hits against the human-coded insult sample.

# Appendix B. Recruitment, inclusion criteria, and sample description

## B1. Eligibility and recruitment logic

Survey respondents were eligible if they had been active in relevant Tieba-related Japanese-culture or Korean-culture communities during the focal controversy windows, had encountered politicized shaming or closely related hostility, and had been adolescents at the time of the focal events.

The study used purposive recruitment to identify theoretically informative cases rather than to estimate population prevalence. The objective was to capture adolescents who had experienced the focal form of politicized public shaming during the relevant periods.

Timeline interviews were drawn from the wider pool using maximum-variation logic so that the three adaptation pathways, support conditions, and management practices were all represented in the qualitative sample.

## B2. Sample summary

**Table B1**

Sample Summary

| **Component** | **Survey** | **Timeline interviews** |
| --- | --- | --- |
| N | 84 | 18 |
| Developmental timing | Adolescents at event exposure; surveyed retrospectively as young adults after the focal windows | Participants reconstructed adolescent experiences retrospectively |
| Age at event exposure | M = 16.06 years, SD = 1.40; range = 14–18 | Exposure ages reconstructed retrospectively from adolescent event windows |
| Women | 63.1% | Mixed by design; not quota-sampled |
| Community coverage | 50 Japan-related; 34 Korea-related | Both community types represented |
| Selection logic | Exposure-relevant purposive sample | Maximum variation across pathways, support, and management practices |
| Analytic role | Primary quantitative sample | Mechanism interpretation and pathway illustration |

Across the combined survey sample (N = 84), mean age at event exposure was 16.06 years (SD = 1.40; range = 14–18). Respondents completed the retrospective survey as young adults after the 2012 and 2016 event windows, corresponding to approximate recall intervals of 11 years for the 2012 cohort and 7 years for the 2016 cohort. The sample was 63.1% women and comprised 50 Japan-related and 34 Korea-related cases.

## B3. Ethics and data minimisation

All survey and interview materials were de-identified prior to analysis. Sensitive identifiers were removed, and examples containing slurs were minimised and masked in reporting. Detailed participant-by-participant listings are omitted here to protect anonymity; the cohort summaries reported below are sufficient for interpreting the present analyses.

# Appendix C. Environmental data, pathway rules, and robustness checks

## C1. Environmental data bundles and front-end retrieval

This appendix documents page retrieval, baseline construction, retrieval success, and the auxiliary environmental model referenced in the main manuscript table notes.

**Table C1**

Environment Data Bundles and Front-End Retrieval

| **Package** | **Contents retained for replication and verification** |
| --- | --- |
| Thread registry and retrieval logs | Target thread list, scraping outcomes, missingness flags, and insufficient-first-screen flags. |
| Page-level measures | Page-level Esem and Evis extraction results and page metadata. |
| Aggregation and overlap | Unit aggregation, baseline-event contrasts, and overlap diagnostics linking respondents to crawled units. |
| Validation bundles | Stigma-lexicon validation sample and first-screen stance-coding reliability materials. |
| Survey datasets | Raw survey export, analysis-ready dataset, and minimal replication outputs. |

## C2. Retrieval success and baseline-event contrasts

**Table C2**

Retrieval Success

| **Period** | **Targeted** | **Retrieved** | **Missing** | **Retrieval rate** | **First-screen partial** | **Partial rate** |
| --- | --- | --- | --- | --- | --- | --- |
| Baseline | 1676 | 1529 | 147 | 91.2% | 60 | 3.6% |
| Event | 1564 | 1343 | 221 | 85.9% | 75 | 4.8% |

*Note.* Pages with fewer than five codable first-screen items were flagged as insufficient first-screen cases according to the pre-specified coding rule.

**Table C3**

Baseline-Event Contrasts for Front-End Indicators

| **Measure** | **Baseline mean** | **Event mean** | **t statistic** | **p value** |
| --- | --- | --- | --- | --- |
| Semantic hostility (Esem) | 0.0037 | 0.0241 | 12.3984 | 1.59e-10 |
| First-screen nationalist dominance (Evis) | 0.6550 | 0.8786 | 8.9011 | 2.89e-10 |

## C3. Selected variable dictionary for the manuscript

**Table C4**

Selected Variable Dictionary for the Manuscript

| **Variable** | **Description** |
| --- | --- |
| Esem_z | Standardised semantic-hostility exposure used in auxiliary models. |
| Evis_z | Standardised first-screen nationalist dominance exposure used in auxiliary models. |
| ICI_index | Raw 1–7 ICI_Total index (13-item mean; full-item robustness version). |
| ICI_expression | Expression-related long-term identity tension subscale. |
| ICI_belonging | Belonging-related long-term identity tension subscale. |
| ICI_selfeval | Self-evaluative long-term identity tension subscale. |
| ICI_reg | Scaled and centred ICI version used in selected robustness models. |
| PS | Peer support composite (combining support seeking and solidarity). |
| VM-B | Behavioural visibility management composite (DIS01–DIS03); operationalises post-event boundary regulation. |
| VM-Cost | Visibility-management cost composite. |
| I1 | Post-event Tieba use-change indicator. |

## C4. Pathway definitions and identification rules

**Table C5**

Pathway Definitions and Identification Rules

| **Pathway** | **Core features** | **Typical practices** | **Identification rule** |
| --- | --- | --- | --- |
| Limited expression | Continued public participation under higher support and higher management | Cautious wording, selective interaction, controlled visibility | VM-B > 4.5 and PS > 4.0 |
| Circle contraction | Higher management under lower support, with interaction relocated into narrower spaces | Alternate accounts, smaller groups, private chats, blocking or diversion | VM-B > 4.5 and PS <= 4.0 |
| Public silence | Durable withdrawal from public expression with lower active management | Reduce or stop public posting; exit topics and sometimes the platform | VM-B <= 4.5 |

*Note.* The pathway labels are analytic classifications rather than latent natural types. Low peer support is not a definitional criterion for public silence; however, in the observed sample this pathway also showed the lowest mean peer support (M = 1.72), which supports the substantive interpretation of durable withdrawal under weak relational support.

**Table C6**

Pairwise Contrasts Across Adaptation Pathways

| **Outcome** | **Comparison** | **Mean diff. [95% CI]** | **Test statistics** |
| --- | --- | --- | --- |
| ICI* (tension) | Public silence vs limited expression | 2.68 [2.30, 3.06] | t(41) = 14.34, p <.001, g = 4.20 |
| ICI* (tension) | Public silence vs circle contraction | 0.85 [0.59, 1.11] | t(34) = 6.76, p <.001, g = 1.58 |
| ICI* (tension) | Circle contraction vs limited expression | 1.83 [1.39, 2.27] | t(39) = 8.33, p <.001, g = 2.31 |
| VM-Cost | Public silence vs limited expression | -1.15 [-1.45, -0.84] | t(38) = -7.58, p <.001, g = -2.15 |
| VM-Cost | Public silence vs circle contraction | -2.74 [-3.04, -2.43] | t(51) = -18.00, p <.001, g = -4.36 |
| VM-Cost | Circle contraction vs limited expression | 1.59 [1.22, 1.96] | t(54) = 8.63, p <.001, g = 2.22 |
| I1 (use change) | Public silence vs limited expression | -1.53 [-1.77, -1.28] | t(48) = -12.56, p <.001, g = -3.48 |
| I1 (use change) | Public silence vs circle contraction | -0.93 [-1.14, -0.72] | t(52) = -9.00, p <.001, g = -2.34 |
| I1 (use change) | Circle contraction vs limited expression | -0.60 [-0.82, -0.38] | t(44) = -5.43, p <.001, g = -1.48 |

*Note.* p values are Benjamini-Hochberg adjusted; g = Hedges' g. These complete pairwise contrasts were moved to the supplement in the compressed manuscript so that the main text could report only the pathway summary and core interpretation.

## C5. Auxiliary environment-indicator model

**Table C7**

Auxiliary Environment-Indicator Model

| **Variable** | **Coefficient** | **SE** | **p** |
| --- | --- | --- | --- |
| Intercept | 0.162 | 0.093 | .083 |
| Esem_z (slur-density exposure) | 0.528 | 0.266 | .047 |
| Evis_z (first-screen nationalist dominance) | 0.320 | 0.249 | .198 |
| R² | 0.503 | — | — |
| N | 84 | — | — |

*Note.* This auxiliary model uses the scaled and centred ICI_reg outcome to show that semantic hostility was positively associated with identity tension even before the main interaction model was considered.

## C6. Construct de-duplication and interaction robustness

**Table C8**

Raw-Scale Interaction Model Predicting ICI*

| **Predictor** | **Model 1 B (SE), p** | **Model 2 B (SE), p** |
| --- | --- | --- |
| Intercept | 5.297 (0.112), p <.001 | 5.277 (0.288), p <.001 |
| Peer support (mean-centred) | -0.680 (0.077), p <.001 | -0.665 (0.088), p <.001 |
| Behavioural visibility management (mean-centred) | 0.337 (0.118), p = .004 | 0.339 (0.136), p = .012 |
| Peer support × visibility management | 0.275 (0.076), p <.001 | 0.281 (0.092), p = .002 |
| Female (= 1) |  | 0.160 (0.212), p = .452 |
| Age at event exposure (z) |  | -0.038 (0.088), p = .665 |
| Bachelor’s degree or above (= 1) |  | -0.081 (0.183), p = .659 |
| Platform tenure (z) |  | -0.072 (0.094), p = .442 |
| Early participation (= 1) |  | -0.045 (0.097), p = .641 |
| National identification strength (z) |  | -0.076 (0.095), p = .425 |
| Event cohort (2016 = 1) |  | 0.146 (0.188), p = .438 |
| Semantic hostility exposure (Esem_z) |  | -0.093 (0.265), p = .726 |
| Visibility pressure exposure (Evis_z) |  | 0.044 (0.242), p = .854 |
| N | 84 | 84 |
| R² | 0.719 | 0.733 |

*Note.* Entries are unstandardised coefficients on the raw 1–7 ICI* scale with HC3 robust standard errors in parentheses. Model 1 includes peer support, behavioural visibility management, and their interaction. Model 2 adds demographic and exposure-related covariates. This is the raw-scale model summarised in the Results section of the main manuscript.

**Table C9**

Construct De-Duplication and Interaction Robustness

| **Specification (z-standardised robustness models)** | **PS B** | **VM-B B** | **PS × VM-B B** | **R²** |
| --- | --- | --- | --- | --- |
| Original scale (ICI_Total; VM = DIS01-DIS06) | -0.598 | 0.454 | 0.356 | 0.783 |
| Exclude ICI12 (ICI*; VM = DIS01-DIS06) | -0.592 | 0.444 | 0.354 | 0.778 |
| Exclude DIS04-DIS06 (ICI_Total; VM = DIS01-DIS03) | -0.687 | 0.345 | 0.277 | 0.722 |
| Double exclusion (ICI*; VM = DIS01-DIS03) | -0.680 | 0.337 | 0.275 | 0.719 |

*Note.* These rows use z-standardised robustness specifications and are included to show sign and significance stability after de-duplication. The raw-scale ICI* model is reported in Table C8 and summarised in the Results section of the main manuscript.

**Table C10**

Factor Pattern for ICI* and VM-B

| **Item** | **Factor 1: ICI*** | **Factor 2: VM-B** |
| --- | --- | --- |
| ICI01 | 0.98 | 0.00 |
| ICI02 | 0.98 | 0.02 |
| ICI03 | 0.98 | -0.07 |
| ICI04 | 0.86 | 0.44 |
| ICI05 | 0.97 | -0.11 |
| ICI06 | 0.77 | 0.17 |
| ICI07 | 0.99 | 0.09 |
| ICI08 | 0.98 | -0.14 |
| ICI09 | 0.98 | 0.01 |
| ICI10 | 0.99 | 0.10 |
| ICI11 | 0.97 | -0.10 |
| ICI13 | 0.99 | 0.09 |
| DIS01 | -0.29 | 0.92 |
| DIS02 | -0.27 | 0.91 |
| DIS03 | -0.47 | 0.85 |

*Note.* The two-factor solution supports discriminant validity between long-term identity tension and behavioural visibility management, while also showing why the overlapping online-offline discrepancy content needed to be separated analytically.

## C7. External validity of pathway labels

**Table C11**

Post-Event Tieba Use by Adaptation Pathway

| **Pathway** | **Completely left** | **Substantially reduced** | **No significant change** | **Within-group n** |
| --- | --- | --- | --- | --- |
| Public silence | 21 (77.8%) | 6 (22.2%) | 0 (0.0%) | 27 |
| Circle contraction | 0 (0.0%) | 28 (84.8%) | 5 (15.2%) | 33 |
| Limited expression | 0 (0.0%) | 6 (25.0%) | 18 (75.0%) | 24 |

*Note.* The I1 cross-tab shows that the labels map onto substantively different post-event platform behaviours rather than merely reflecting threshold placement.

## C8. Threshold perturbation and comparison with unsupervised clustering

**Table C12**

Threshold Perturbation and Clustering Comparison

| **Scenario** | **VM-B threshold** | **PS threshold** | **Group sizes (PS / CC / LE)** | **ARI** |
| --- | --- | --- | --- | --- |
| Baseline threshold | 4.5 | 4.0 | 27 / 33 / 24 | 1.00 |
| VM-B threshold = 4.0 | 4.0 | 4.0 | 27 / 33 / 24 | 1.00 |
| PS threshold = 3.5 | 4.5 | 3.5 | 27 / 33 / 24 | 1.00 |
| PS threshold = 4.5 | 4.5 | 4.5 | 27 / 33 / 24 | 1.00 |
| VM-B threshold = 5.0 | 5.0 | 4.0 | 35 / 28 / 21 | 0.72 |
| k-means (k = 3, standardised PS and VM-B) | — | — | Consistent with baseline | 1.00 |

## C9. Alternative operationalisations of peer support

**Table C13**

Alternative Operationalisations of Peer Support

| **PS operationalisation** | **PS main effect** | **VM-B main effect** | **Interaction** | **R²** |
| --- | --- | --- | --- | --- |
| PS mean of three items | -0.665 (0.088) | 0.339 (0.136) | 0.281 (0.092) | 0.733 |
| Support received only (CS01) | -0.646 (0.087) | 0.353 (0.139) | 0.265 (0.088) | 0.732 |
| Solidarity / standing together only (mean of CS02-CS03) | -0.671 (0.089) | 0.328 (0.134) | 0.287 (0.094) | 0.731 |

*Note.* The direction and magnitude of the interaction were stable whether PS was treated as a combined peer-support composite or split into its two substantive components.

## C10. Distribution and ceiling checks

**Table C14**

Distribution and Ceiling Checks

| **Group** | **n** | **ICI* M (SD)** | **ICI* %max** | **IMP M (SD)** | **IMP %max** | **PS M (SD)** | **VM-B M (SD)** |
| --- | --- | --- | --- | --- | --- | --- | --- |
| Public silence | 27 | 6.67 (0.17) | 48.1% | 5.00 (0.00) | 100.0% | 1.72 (0.45) | 3.38 (0.37) |
| Circle contraction | 33 | 5.83 (0.69) | 0.0% | 4.45 (0.51) | 45.5% | 2.41 (0.40) | 6.24 (0.68) |
| Limited expression | 24 | 3.99 (0.90) | 0.0% | 2.92 (0.72) | 0.0% | 5.99 (0.73) | 5.81 (0.47) |
| Full sample | 84 | 5.57 (1.25) | 15.5% | 4.19 (0.98) | 50.0% | 3.21 (1.86) | 5.20 (1.38) |

*Note.* The public-silence group showed clear ceiling concentration, especially for IMP. These concentrations are reported so that the contrasts can be interpreted as strongly directional and substantively important, while avoiding overstatement about scale-linear magnitudes. Accordingly, IMP is summarised descriptively rather than tested with parametric group contrasts in the main manuscript.
